# Active pathways of anaerobic methane oxidation across contrasting riverbeds

**DOI:** 10.1038/s41396-018-0302-y

**Published:** 2018-10-30

**Authors:** Li-dong Shen, Liao Ouyang, Yizhu Zhu, Mark Trimmer

**Affiliations:** 1 Collaborative Innovation Center on Forecast and Evaluation of Meteorological Disasters, Jiangsu Key Laboratory of Agricultural Meteorology, School of Applied Meteorology, Nanjing University of Information Science and Technology, Nanjing, 210044 China; 2 School of Biological & Chemical Sciences, Queen Mary University of London, London, E1 4NS UK

**Keywords:** Environmental microbiology, Microbial ecology

## Abstract

Anaerobic oxidation of methane (AOM) reduces methane emissions from marine ecosystems but we know little about AOM in rivers, whose role in the global carbon cycle is increasingly recognized. We measured AOM potentials driven by different electron acceptors, including nitrite, nitrate, sulfate, and ferric iron, and identified microorganisms involved across contrasting riverbeds. AOM activity was confined to the more reduced, sandy riverbeds, whereas no activity was measured in the less reduced, gravel riverbeds where there were few anaerobic methanotrophs. Nitrite-dependent and nitrate-dependent AOM occurred in all sandy riverbeds, with the maximum rates of 61.0 and 20.0 nmol CO_2_ g^−1^ (dry sediment) d^−^^1^, respectively, while sulfate-dependent and ferric iron-dependent AOM occurred only where methane concentration was highest and the diversity of AOM pathways greatest. Diverse *Candidatus* Methylomirabilis oxyfera (*M. oxyfera*)-like bacteria and *Candidatus* Methanoperedens nitroreducens (*M. nitroreducens*)-like archaea were detected in the sandy riverbeds (16S rRNA gene abundance of 9.3 × 10^5^ to 1.5 × 10^7^ and 2.1 × 10^4^ to 2.5 × 10^5^ copies g^−^^1^ dry sediment, respectively) but no other known anaerobic methanotrophs. Further, we found *M. oxyfera*-like bacteria and *M. nitroreducens*-like archaea to be actively involved in nitrite- and nitrate/ferric iron-dependent AOM, respectively. Hence, we demonstrate multiple pathways of AOM in relation to methane, though the activities of *M. oxyfera*-like bacteria and *M. nitroreducens*-like archaea are dominant.

## Introduction

Anaerobic oxidation of methane (AOM) via sulfate reduction reduces methane emissions from marine sediments [1]. Sulfate-dependent AOM is catalyzed by anaerobic methanotrophic archaea of the ANME-1, ANME-2a/2b, ANME-2c, and ANME-3 clades and by sulfate-reducing bacteria. The transfer of  reducing equivalents to sulfate is through an interspecies electron carrier or conductive pili (nanowires) [2–4], or by excreting zero-valent sulfur compounds for disproportionation by their bacterial partners [5].

Methane can also be oxidized anaerobically using alternative electron acceptors, e.g., nitrite [6], nitrate [7] and ferric iron [8, 9]. Nitrite-dependent AOM is performed by the NC10 bacteria related to *Candidatus* Methylomirabilis oxyfera (*M. oxyfera*) [6, 10], that produces oxygen intracellularly from two nitric oxide molecules for methane oxidation and respiration [10, 11]. A specific lineage of the ANME-2 clade (ANME-2d) (*Candidatus* Methanoperedens nitroreducens [*M. nitroreducens*]) is capable of catalyzing AOM through reverse methanogenesis using nitrate [7]. Ettwig et al. [8] showed that *M. nitroreducens*-like archaea can oxidize methane using ferric iron in addition to nitrate. Very recently, Cai et al. [9] reported a novel genus within the family *Candidatus* Methanoperedenaceae that  can also couple methane oxidation to ferric iron reduction. All these anaerobic methanotrophs have been enriched from various freshwater sediments.

Our knowledge about sulfate-dependent AOM is mainly for marine sediments [12–14], as it was assumed to be insignificant in low-sulfate fresh waters (~0.01–0.2 mM versus 28 mM in sea water) [15]. Some have suggested, however, that AOM is coupled to sulfate reduction in lake sediments [16–18]. Recently, Weber et al. [19] provided direct evidence that the *M. nitroreducens*-like archaea are involved in sulfate-dependent AOM in lake sediments through 16S rRNA-based stable isotope probing. Studies of nitrite-dependent AOM in fresh waters are increasing, including wetlands [20, 21], lakes [22–24], and reservoirs [25, 26]. Nitrate-dependent AOM could also reduce methane emissions from wetlands [21] and paddy fields [27, 28]. Due to these more diverse electron acceptors in fresh waters, they may support a greater variety of AOM pathways than marine sediments.

Contemporary analyses have increased the estimated contribution from running waters (rivers and streams) to the global methane budget from 1.5 to 26.8 Tg CH_4_ per year, equivalent to ~15 and 40% of emissions from wetlands and lakes, respectively [29, 30]. Further, our previous work demonstrated that a rise of just 2 °C could increase the proportion of carbon emitted as methane from rivers by 8% [31]. Thus rivers could play an increasing role in the future global methane budget, and our appreciation of rivers as biogeochemical hotspots and major contributors to the global carbon cycle is being revised.

Aerobic methane oxidation reduces methane emissions from rivers [31, 32], but anaerobic oxidation via nitrite, nitrate, sulfate, and ferric iron cannot be excluded. Only molecular evidence for AOM (presence of *M. oxyfera*-like sequences) has hitherto been reported for rivers [33–35], although nitrite-dependent AOM activity was discovered in an enrichment culture from canal sediments [6]. Overall, the potential of different AOM pathways to reduce methane emissions from rivers, the microorganisms involved, are poorly known.

Many of the lake and wetland sediments known to support AOM are probably impermeable silts and clays [20, 21, 23], where diffusion delivers the solutes that sustain microbial metabolism [36]. In contrast, the gravel and sandy sediments which dominate UK riverbeds [37] are more permeable [38, 39], allowing greater advective flux of nutrients (inorganic nitrogen and phosphorus etc.,) and methane through the riverbed. Even though the bulk porewater of such riverbeds has appreciable oxygen, anaerobic metabolism (e.g., anammox and denitrification) can occur in anoxic microsites [39, 40]. The physical heterogeneity of the riverbeds and the co-occurrence of methane and nitrate reduction, along with other electron acceptors [39, 41], could allow the co-existence of various AOM communities. Further, the contrasting characteristics of gravels and sands (e.g., the porewater methane concentration in sands is far greater than that in gravels [39, 42]) in turn, could affect the distribution and activity of AOM communities.

Our aim was to characterize the significance of AOM pathways driven by the electron acceptors nitrite, nitrate, sulfate, and ferric iron in reducing methane emissions, and to identify the anaerobic methanotrophs actively involved along a gradient of declining methane concentration from sandy to gravel riverbeds.

## Materials and methods

### Sample collection

**Fig. 1:**
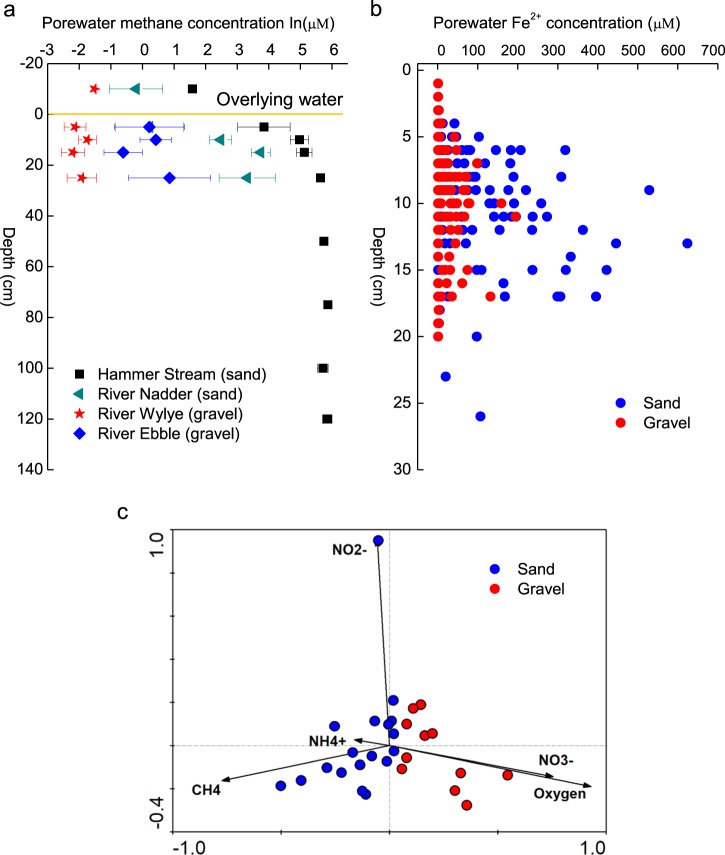
Porewater **a** methane (ln, natural logarithm) and **b** Fe^2+^ concentrations across contrasting sandy and gravel riverbeds (data from ref. [41]; and unpublished from ref. [39], which are available at 10.5285/7ded510f-3955- 4b92-851d-29c0f79a0b99) and **c** PCA ordination diagram of porewater chemisty within our present riverbeds

Sediment and porewater were collected from seven riverbeds in southeast England between February 2016 and January 2017 (Table S1). The rivers Lambourn and Stour (I and II) have predominantly gravel beds (grain size 2–16 mm) and overlie permeable chalk geology, while the Hammer Stream and rivers Medway, Marden, and Nadder have predominantly sandy beds (grain size 0.062–2 mm) which are typically less permeable [43, 44]. In each river, six intact sediment cores were collected to a depth of 16 cm and porewaters (five replicates) sampled using mini-probes [38]. Porewater samples for NH_4_^+^, NO_2_^−^, and NO_3_^−^ analyses were preserved by filtering (0.2 μm polypropylene) and samples for methane (CH_4_) transferred to vials (3 ml, Labco, UK) and preserved with ZnCl_2_ (100 μl, 50% (w/v)). Surface waters were treated in the same way and all samples were kept in cool bags (Thermos) for transfer to the laboratory (<4 h). Sediment cores were then sliced in an anoxic glove box (AGB) (O_2_ < 100ppm; Belle Technology, Dorset, UK) at five depths horizons: 0–2, 2, 4–8, 8–12, and 12–16 cm. This study is supported in part by published methane and Fe^2+^ data (Fig. 1a, b) generated by a study of in situ nitrogen cycling in the Hammer Stream [41] and a wider study of the biogeochemistry in rivers on chalk, sand, and clay geologies [39, 43, 45].

### Physicochemical analyses

Surface waters and porewaters were transferred through a three-way stopcock into a syringe holding a pH or O_2_ probe [38] and the concentrations of NH_4_^+^, NO_2_^−^, and NO_3_^−^ measured using an auto-analyzer (Skalar San^+2^, Breda, The Netherlands). Methane concentration was measured by gas chromatography (GC/FID), as described previously [31].

### Isotope tracer experiments

AOM activity was measured using ^13^CH_4_. Approximately 2−3 g of sediments and 5 ml of N_2_-degassed synthetic river water (0.12 g l^−^^1^ NaHCO_3_, 0.04 g l^−^^1^ KHCO_3_, 0.027 g l^−^^1^ MgCl_2_, and 0.092 g l^−^^1^ CaCl_2_·2H_2_O) were transferred into 12 ml vials (Labco, UK) inside the AGB and then preincubated for 2d on orbital shakers to remove background NO_*x*_^−^ (NO_2_^−^ + NO_3_^−^). After preincubation, the vials were injected with 100 μl of N_2_-degassed stock solutions of either NaNO_2_ (1 mM), NaNO_3_ (2.5 mM), FeCl_3_ (1 mM) or Na_2_SO_4_ (1 mM). Finally, 50 μl of ^13^CH_4_ was injected to give a ~1% (v/v) of methane headspace and control slurries were left unamended. Our incubations to measure AOM potential lasted less than 4 weeks, during which six replicate vials were sacrificed (300 μl, ZnCl_2_, 50% (w/v)) at different intervals and headspace concentrations of ^12^CO_2_ and ^13^CO_2_ (*m*/*z* 44 and 45) measured using CF/IRMS. The overlying water in each vial was then collected and acidified (HCl, 100 μl, 12.2 M), and DIC (CO_2_ + HCO_3_^−^ + CO_3_^2−^) measured as ^12^CO_2_ or ^13^CO_2_ [32], where ^13^CO_2_ plus ^13^C-DIC equalled the total amount of ^13^C-CH_4_ oxidized to inorganic carbon. An obvious lag phase was observed before ^13^CO_2_ production in slurries amended with ^13^CH_4_ + NO_2_^−^ or NO_3_^−^ (Results section), therefore AOM activity was calculated by linear regression after the lag phase. Having demonstrated a potential for AOM, we then repeated the incubations using a shorter preincubation period (6 h) to see if we could remove any lag phase and measure the potentials closer to in situ (Results section).

To determine organic ^13^C content, the incubated sediments were acidified (as above) to remove DIC, dried to a constant mass (60 °C) and then combusted at 1000 °C in an integrated elemental analyzer and mass spectrometer (Sercon Integra 2). The AOM carbon conversion efficiency (CCE) was calculated as the fraction of total ^13^C-CH_4_ oxidized to organic carbon.

### DNA extraction

Genomic sediment DNA was extracted using a Power Soil DNA Isolation Kit (MO-BIO-Laboratories, USA) and its concentration and quality measured with a NanoDrop (ND-1000; Isogen Life Science, the Netherlands).

### PCR amplification and Illumina Miseq sequencing

The hypervariable regions of the bacterial (V3–V4) and archaeal (V4–V5) 16S rRNA genes were amplified with the universal primer sets 319f-806r [46] and 524f-958r [47], respectively. The preparation of the PCR mixtures and thermal programmes for the bacteria and archaea were as described previously [21, 48]. Total bacterial and archaeal PCR products were gel purified using QIAquick gel extraction kits (Qiagen, Chatsworth, California, USA) and sequenced using the 300 bp paired-end strategy on the Illumina MiSeq platform. The downstream sequence analyses were conducted using the Quantitative Insights into Microbial Ecology (QIIME) [49]. The reads from the original DNA fragments were merged, and reads with an average quality score above 25 were accepted. The occurrence of chimeric sequences was further examined using QIIME. High-quality reads were then classified with the Ribosomal Database Project (RDP) classifier using the SILVA databases.

### Phylogenetic analysis

Phylogenetic analysis of the sequences was performed using Mega 5 software; evolutionary distance computed using the Maximum Likelihood and tree topology tested using bootstrap analysis (1000 replicates).

### Quantitative PCR

The 16S rRNA gene abundance of total bacteria and total archaea was determined using the primer sets 341f-518r [50] and Arch967f-Arch1060r [51], respectively. Primer sets of qp1f-qp1r [52] and 641F-834R [17] were used for quantification of the 16S rRNA genes of *M. oxyfera*-like bacteria and *M. nitroreducens*-like archaea, respectively (see Table S1 for details of quantitative PCR (qPCR) primers). The qPCR was performed on an iCycler iQ5 thermocycler (BioRad) with standard curves constructed from a serial dilution of a known copy number of the plasmid DNA. Triplicate qPCRs analyses were performed for each sample and each dilution and the specialties of qPCR products examined by melt curve analysis with a detection limit of 9.2–10.8 copies per well.

### Real-time quantitative reverse transcription PCR

Total RNA was extracted using a RNA PowerSoil Total RNA Isolation Kit (Qiagen, USA) and treated with a DNA-free™ DNA Removal Kit (Invitrogen, USA). RNA concentration was quantified with a Qubit 2.0 Fluorometer (Invitrogen, USA) and total RNA then used for cDNA synthesis using Super-ScriptTM II reverse transcriptase (Invitrogen, USA). Transcript abundance of *M. oxyfera*-like bacterial *pmo*A mRNA and *M. nitroreducens*-like archaeal *mcr*A mRNA were quantified in conjunction with the tracer experiments using the primer sets cmo182-cmo568 [53] and McrA159f-McrA345r [35], respectively. The RT-qPCR was conducted as for qPCR described above using BioRad CFX 384, with a detection limit of 9.8–10.7 copies per well.

## Results

### Porewater chemistry

Porewater methane in sandy riverbeds (0.7–354.3 μM) was significantly higher (*t*-test, *p* < 0.05) than that in the gravels (0.02–34.0 μM), and tended to increase with depth (Fig. S1). In contrast, oxygen was significantly lower (*t*-test, *p* < 0.05) in the sand (38.7–133.3 μM) compared to the gravel (92.6–288.7 μM) (Fig. S1) and, overall, the sandy Hammer Stream had the highest methane with the lowest oxygen. Porewater nitrite was 0.5–5.9 μM across the riverbeds, and was highest in the upper 2 cm sediments (Fig. S1). Nitrate concentration decreased with depth in the sand (8–116 μM) but increased with depth in the gravel (57–489 μM), reflecting the stronger influence of groundwater on the more permeable chalk. Ammonium concentration was low in the Lambourn (0.7–1.2 μM) but extremely high in the Medway (476.0–2354.4 μM) (Fig. S1). Elsewhere ammonium concentration was 7.9–184.5 μM. Shelley et al. [41] reported mean Fe^2+^ concentrations in the Hammer Stream sediments of 178 μM, and data generated by Lansdown et al. [39] clearly show higher concentrations for Fe^2+^ in sandy riverbeds (136.9 μM) than in gravels (17.0 μM) (Fig. 1b). According to the present and previously reported data (Table 1), we define sandy riverbeds as being more reduced than gravels, with on average about 10 times more methane, 8 times more Fe^2+^, and 50% less oxygen. Further, principle component analysis (PCA) of our present riverbeds showed sharp separation of oxygen and methane on PC1 (accounted for 59% of the variance, Fig. 1c), suggesting that this axis represented a chemical gradient moving from more reduced sandy riverbeds to less reduced gravel riverbeds.

**Table 1:** Summary of the mean values of porewater CH_4_ concentrations, Fe^2+^ concentrations, O_2_ concentrations, and NO_3_^−^ concentrations in the upper 20 cm of gravel and sandy riverbeds and the methane oxidation pathways reported here and previously [39, 41, 64]

Rivers	Bed-types	CH_4_ (μM)	Fe^2+^ (μM)	O_2_ (μM)	NO_3_^−^ (μM)	Electron acceptor(s) for methane oxidation^a^
Stour I	Gravel	11.9	ND	125.6	132.9	O_2_
Stour II	Gravel	18.1	ND	131.8	70.1	O_2_
Lambourn	Gravel	0.03	ND	251.9	462.9	O_2_
Ebble	Gravel	1.1	26.0	53.7	120.8	O_2_
Wyle	Gravel	0.14	7.9	67.7	217.9	O_2_
Hammer Stream	Sand	151.7	178.0	39.1	14.2	O_2_, NO_2_^−^, NO_3_^−^, Fe^3+^, SO_4_^2−^
Medway	Sand	49.1	ND	85.6	73.4	O_2_, NO_2_^−^, NO_3_^−^
Marden	Sand	12.8	ND	74.6	68.7	O_2_, NO_2_^−^, NO_3_^−^
Nadder	Sand	58.4	136.9	73.9	38.7	O_2_, NO_2_^−^, NO_3_^−^

*ND* not determined

^a^NO_3_^−^ could be used directly in AOM or after reduction to NO_2_^−^

### AOM potential driven by different electron acceptors

**Fig. 2:**
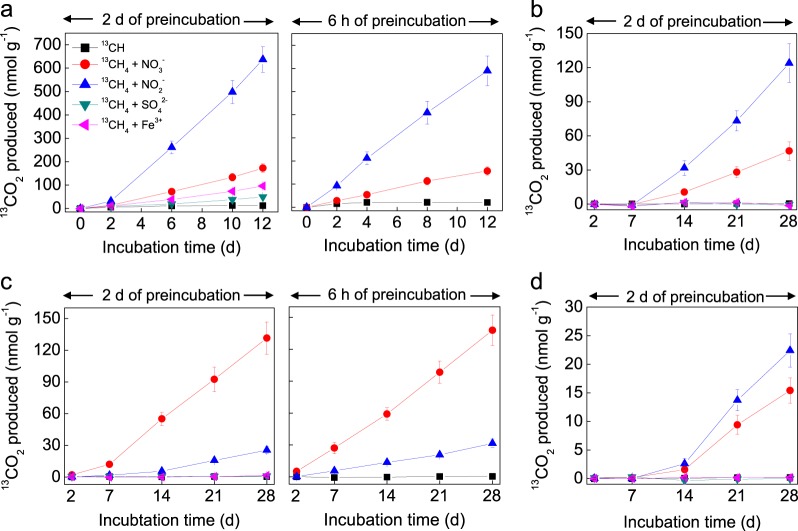
Examples of ^13^CO_2_ production in slurries of sediment from the 4 to 8 cm depth horizon amended with ^13^CH_4_ and different electron acceptors after both short and long preincubations. **a** and **c** both durations in the Hammer stream and River Nadder, respectively, and **b** and **d** long preincubations only for the rivers Medway and Marden, respectively

**Fig. 3:**
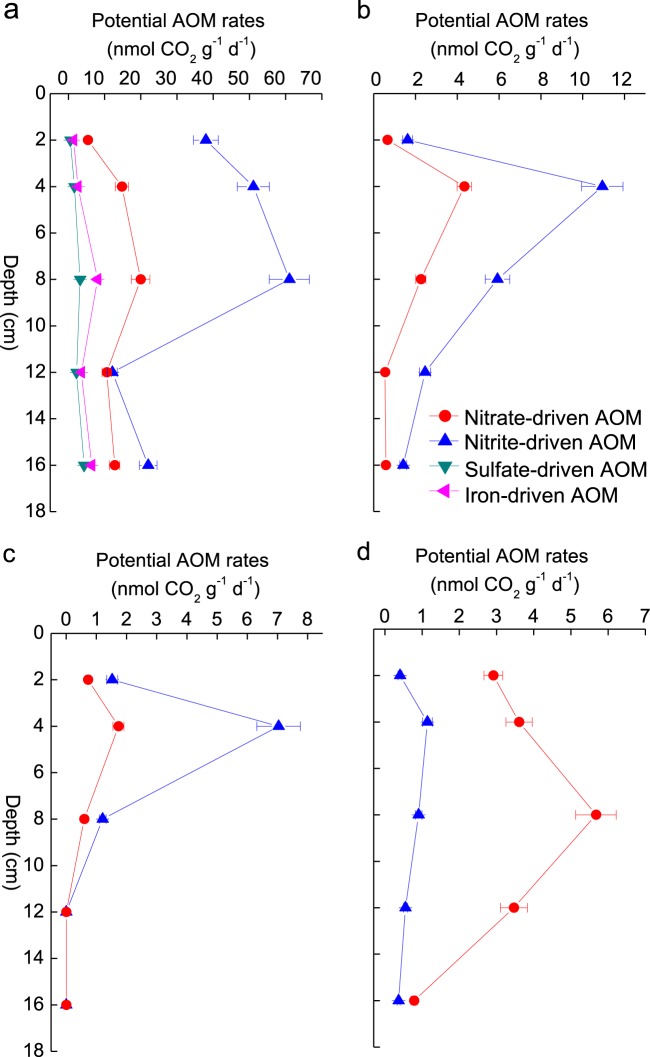
Vertical profiles of the activities of different AOM processes driven by nitrite, nitrate, sulfate and ferric iron in the **a** Hammer Stream, **b** River Medway, **c** River Marden, and **d** River Nadder

The ^13^CH_4_ tracer experiments showed no significant production of ^13^CO_2_ in any of the gravel sediments amended with any electron acceptor (NO_2_^−^, NO_3_^−^, SO_4_^2−^, Fe^3+^) (Fig. S2). In contrast, both NO_2_^−^ and NO_3_^−^ generated ^13^CO_2_ with all sandy sediments; though there was an obvious lag phase before ^13^CO_2_ production began (Fig. 2). Different ranges of nitrite-dependent AOM activity were observed (Fig. 3): the potential in the Hammer Stream (12.2–61.0 nmol CO_2_ g^−^^1^ [dry sediment] d^−^^1^) was significantly higher (*t*-test, *p* < 0.05) than that measured in the Medway, Marden, and Nadder, with activities of 1.4–10.9, 1.2–7.0, and 0.4–1.1 nmol CO_2_ g^−^^1^ (dry sediment) d^−^^1^, respectively. Nitrate-dependent AOM activity was lower than that of nitrite-dependent AOM, except for the River Nadder (Fig. 3). Nitrate-dependent AOM activities were 5.4–20.0, 0.5–4.3, 0.6–1.7, and 0.8–5.7 nmol CO_2_ g^−^^1^ (dry sediment) d^−^^1^, respectively, in the Hammer Stream and rivers Medway, Marden, and Nadder. Having shown the AOM potentials driven by nitrite and nitrate with a lag phase, we collected more sediment from two representative rivers (Hammer Stream and River Nadder) for a second set of experiments with a shorter, 6 h preincubation. Here, there was no lag phase for NO_*x*_^−^-dependent AOM and the activity was similar to that after a 2d preincubation (Fig. 2).

The addition of both SO_4_^2−^and Fe^3+^ stimulated ^13^CO_2_ production in the Hammer Stream (Fig. 2; Table 1). Sulfate-dependent and ferric iron-dependent AOM activities were 0.6–4.4 and 1.5–8.1 nmol CO_2_ g^−^^1^ (dry sediment) d^−^^1^, respectively, at different depths in the riverbed.

Organic ^13^C could only be measured in slurries amended with ^13^CH_4_ + NO_2_^−^ in sediment from the Hammer Stream, where the CCE was 8.2% on average. Therefore, although all the sandy riverbeds exhibited an AOM potential, only the most reduced Hammer Stream provided evidence for a growing AOM community.

### Expression analyses of pmoA mRNA and mcrA mRNA

After 4 weeks of incubation we assayed sediments from the Hammer Stream (4–8 cm layer), both with and without additions of ^13^CH_4_, ^13^CH_4_ + NO_2_^−^, ^13^CH_4_ + NO_3_^−^, ^13^CH_4_ + SO_4_^2−^, and ^13^CH_4_ + Fe^3+^, for gene expression. Similarly, sediments from the River Nadder (2−4 cm layer) were also screened for expression but only in the incubations with ^13^CH_4_ + NO_2_^−^ and ^13^CH_4_ + NO_3_^−^, as only they had produced measurable amounts of ^13^CO_2_.

**Fig. 4:**
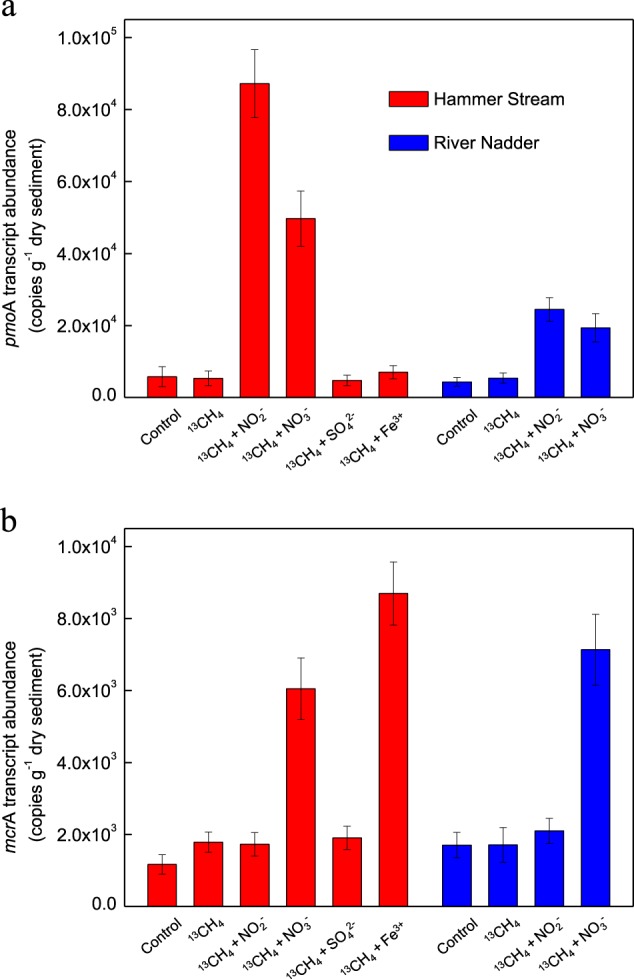
The abundance of **a** *pmo*A transcripts involved in *M. oxyfera*-like bacteria and **b** *mcr*A transcripts involved in *M. nitroreducens*-like archaea in different slurries (after 4 weeks of incubation) with different amendments in the Hammer Stream and River Nadder

The abundances of *pmo*A transcripts in slurries amended with ^13^CH_4_ + NO_2_^−^ (8.7 × 10^4^ copies g^−^^1^ dry sediment) and ^13^CH_4_ + NO_3_^−^(4.9 × 10^4^ copies g^−^^1^ dry sediment) were higher (*t*-test, *p* < 0.05) than those amended with ^13^CH_4_, ^13^CH_4_ + SO_4_^2−^, ^13^CH_4_ + Fe^3+^, and controls (4.6–7.0 × 10^3^ copies g^−^^1^ dry sediment) in the Hammer Stream (Fig. 4a). Similarly, in the River Nadder (Fig. 4a), higher abundances (*t*-test, *p* < 0.05) of *pmo*A transcripts were observed in slurries amended with ^13^CH_4_ + NO_2_^−^ (2.4 × 10^4^ copies g^−^^1^ dry sediment) and ^13^CH_4_ + NO_3_^−^(1.9 × 10^4^ copies g^−^^1^ dry sediment), than in those amended with only ^13^CH_4_ and the controls (4.2–5.4 × 10^3^ copies g^−^^1^ dry sediment).

In the Hammer Stream (Fig. 4b), the abundances of *M. nitroreducens*-like archaeal *mcr*A transcripts were higher (*t*-test, *p* < 0.05) in slurries amended with ^13^CH_4_ + NO_3_^−^ and ^13^CH_4_ + Fe^3+^ (6.0 and 8.7 × 10^3^ copies g^−^^1^ dry sediment, respectively) than in the other treatments or controls (1.2–1.9 × 10^3^ copies g^−^^1^ dry sediment). In the River Nadder, higher (*t*-test, *p* < 0.05) transcript abundance of *mcr*A was also detected, but only in slurries amended with ^13^CH_4_ + NO_3_^−^ (7.1 × 10^3^ copies g^−^^1^ dry sediment), compared to the other treatments and controls (1.7–2.1 × 10^3^ copies g^−^^1^ dry sediment).

Further, the *pmo*A cDNA derived from slurries amended with ^13^CH_4_ + NO_2_^−^ and ^13^CH_4_ + NO_3_^−^, and for *mcr*A in slurries amended with ^13^CH_4_ + NO_3_^−^ and ^13^CH_4_ + Fe^3+^, was cloned and sequenced. Phylogenetic analysis showed that the *pmo*A cDNA (26 sequences) formed a distinct cluster (>99% identity between each other) in the tree, showing distant identity (89.4–90.5%) to the *pmo*A gene of *Candidatus* Methylomirabilis oxyfera [10] but a higher identity (97–99%) to the *pmo*A genes reported for reservoir sediments (Fig. S3). Three different clusters of the *mcr*A cDNA (18 sequences) were present in the phylogenetic tree (Fig. S4), showing 92.5–95.2 and 93.0–98.9% identity to the *mcr*A genes of *Candidatus* Methanoperedens nitroreducens (JMIY0100002) [7] and *Candidatus* Methanoperedens sp. BLZ1 [54], respectively. The three clusters were also closely related (>95% identity) to the *mcr*A genes reported in paddy soils [27] and river sediment [35].

### Bacterial and archaeal communities across the riverbeds

The microbial community composition at different depths of the sandy riverbeds, in which AOM activity was detected, was examined by Illumina sequencing of the total bacterial and archaeal 16S rRNA genes. After tag merge, removal of chimeric sequences and quality control, the ranges of total (clean) bacterial and archaeal sequences were 42,505–64,875 and 30,287–42,758, respectively (Table 2).

**Table 2:** The percentages of sequences related to *M. oxyfera*-like bacteria and *M. nitroreducens*-like archaea

Sediment samples	No. of clean bacterial sequences	Percentage of *M. oxyfera*-like sequences (%)	No. of clean archaeal sequences	Percentage of *M. nitroreducens*-like sequences (%)
Hammer stream (2 cm)	42,873	0.8%	34,889	1.5%
Hammer stream (4 cm)	44,753	2.1%	37,535	2.7%
Hammer stream (8 cm)	56,285	1.4%	30,287	2.4%
Hammer stream (12 cm)	46,346	1.2%	30,701	2.1%
Hammer stream (16 cm)	58,420	0.8%	35,327	2.3%
River Medway (2 cm)	55,650	0.5%	42,758	0.06%
River Medway (4 cm)	53,801	0.9%	40,285	0.9%
River Medway (8 cm)	59,960	0.4%	37,752	0.4%
River Medway (12 cm)	63,673	0.3%	41,014	0.7%
River Medway (16 cm)	64,649	0.5%	42,393	0.4%
River Marden (2 cm)	57,741	0.3%	37,840	0.04%
River Marden (4 cm)	49,447	0.8%	34,167	0.05%
River Marden (8 cm)	53,570	0.9%	36,376	0.02%
River Marden (12 cm)	43,728	0.5%	32,415	0.02%
River Marden (16 cm)	64,682	0.8%	34,310	0.03%
River Nadder (2 cm)	64,875	0.6%	34,645	2.2%
River Nadder (4 cm)	51,027	0.4%	38,941	6.3%
River Nadder (8 cm)	60,625	0.7%	37,210	6.5%
River Nadder (12 cm)	42,504	0.3%	37,213	4.5%
River Nadder (16 cm)	43,049	0.5%	40,287	3.3%

Proteobacteria was the most abundant phylum across all riverbeds (Fig. S5). The percentage of *M. oxyfera*-like sequences in the Hammer Stream (0.8–2.1%) was higher than that in other riverbeds (0.3–0.9%; Table 2). The taxonomic affiliations of the sequences for Type I and Type II aerobic methanotrophs were compared at the order, family and genus levels [55, 56] which showed that they comprised <0.1% of the total reads. No sequences related to Verrucomicrobia methanotrophs (*Methylacidiphilum*) [57] could be detected.

The phylum Bathyarchaeota was the dominant archaea in the Hammer Stream and the rivers Medway and Nadder (Fig. S6). In the River Marden, Thaumarchaeota was the dominant phylum. A relatively higher occurrence of *M. nitroreducens*-like sequences was found in the Hammer Stream and River Nadder, accounting for 1.5–2.7 and 2.2–6.5%, respectively, of the total archaeal sequences in each sample (Table 2). No members of the marine anaerobic methanotrophs (ANME-1, ANME-2a/2b, ANME-2c, and ANME-3) were detected.

### Phylogenetic analyses of known anaerobic methanotrophs

**Fig. 5:**
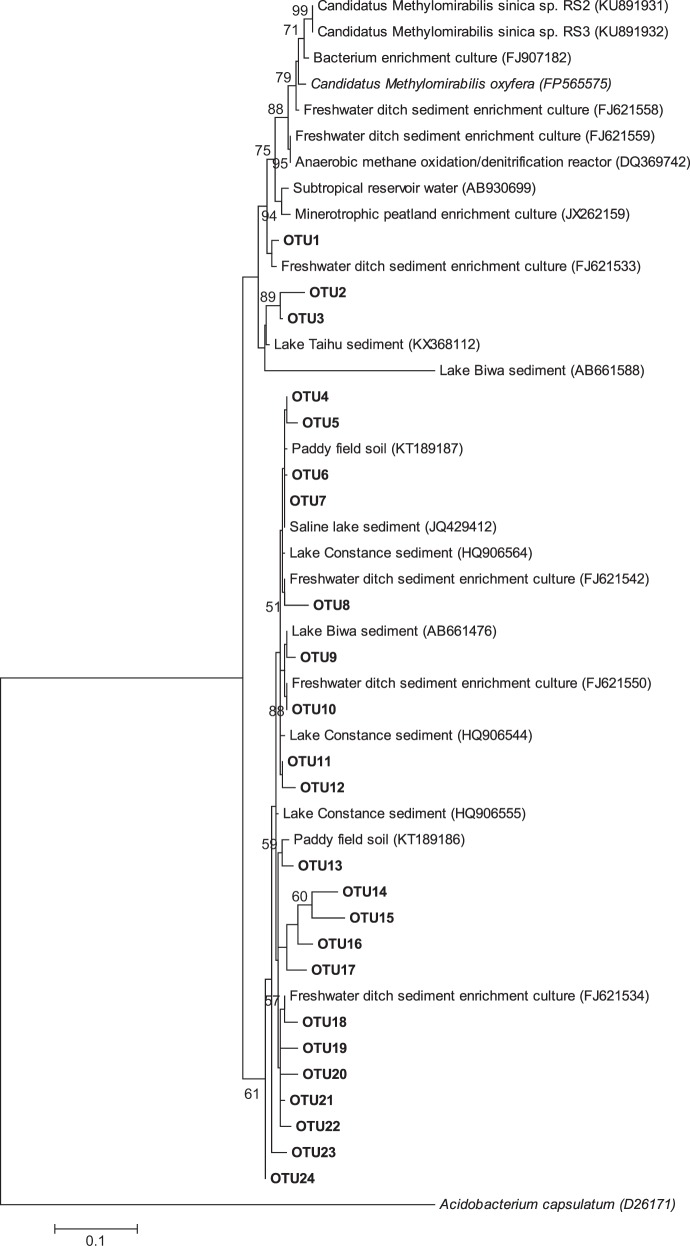
Maximum likelihood phylogenetic tree showing the affiliations of the 16S rRNA genes of *M. oxyfera*-like bacteria (430 bp) recovered from the sandy riverbeds. Bootstrap values > 50% (out of 1000 replicates) are shown in front of respective nodes, and the scale bar represents 10% sequence divergence

Phylogenetic analysis showed that the *M. oxyfera*-like sequences recovered were 90–97% identical to the 16S rRNA gene of *Candidatus* Methylomirabilis oxyfera [10]. These sequences were closely related (>96% identical) to the *M. oxyfera*-like sequences reported in enrichment cultures [52], lake sediments [23, 58, 59], and paddy soil [27] (Fig. 5).

**Fig. 6:**
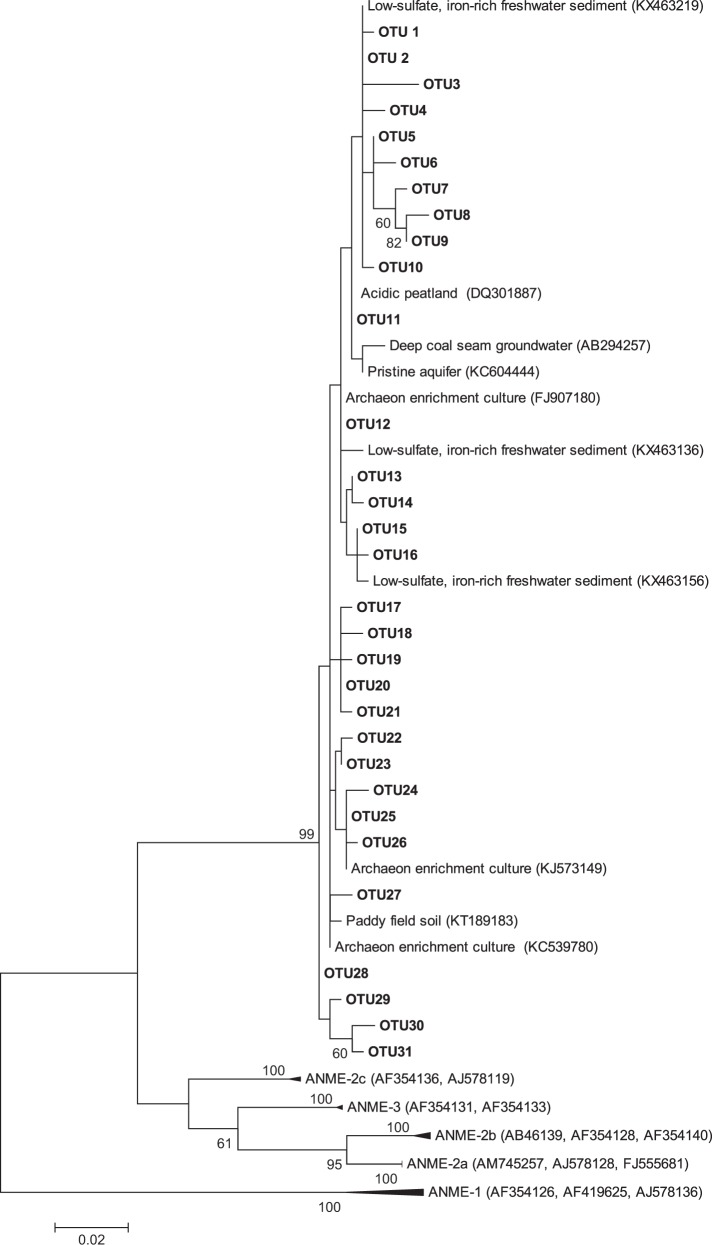
Maximum likelihood phylogenetic tree showing the affiliations of the 16S rRNA genes of *M. nitroreducens*-like archaea (449 bp) recovered from the sandy riverbeds. Bootstrap values > 50% (out of 1000 replicates) are shown in front of respective nodes, and the scale bar represents 2% sequence divergence

Phylogenetic analysis of *M. nitroreducens*-like sequences showed that they were closely related (>96% identical) to the *M. nitroreducens*-like sequences from enrichment cultures [60, 61], lake sediments [19], and paddy soils [27] (Fig. 6).

### Quantification of known anaerobic methanotrophs

**Fig. 7:**
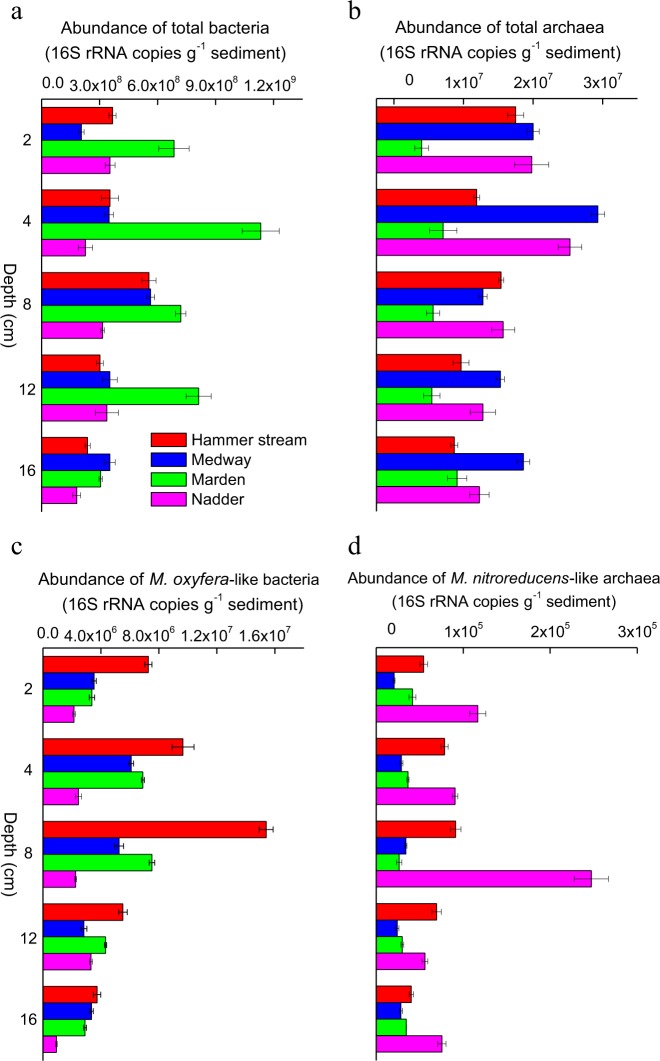
Vertical distribution of the 16S rRNA gene abundance of **a** total bacteria, **b** total archaea, **c** *M. oxyfera*-like bacteria, and **d** *M. nitroreducens*-like archaea in the sandy riverbeds

The 16S rRNA gene abundance of total bacteria (1.8 × 10^8^ to 1.1 × 10^9^ copies g^−^^1^ dry sediment) was higher (*t*-test, *p* < 0.05) than that of archaea (4.0 × 10^6^ to 2.9 × 10^7^ copies g^−^^1^ dry sediment) in all of the riverbeds (Fig. 7). The highest 16S rRNA gene abundance of *M. oxyfera*-like bacteria was detected in the Hammer Stream (3.7 × 10^6^ to 1.5 × 10^7^ copies g^−^^1^ dry sediment). Very low *M. oxyfera*-like bacterial 16S rRNA gene abundance was measured in the gravel riverbeds (<8.7 × 10^3^ copies g^−^^1^ dry sediment). The 16S rRNA gene abundance of *M. oxyfera*-like bacteria correlated well with the potential nitrite-dependent AOM rates (Fig. S7). The ratios of gene abundance of *M. oxyfera*-like bacteria to total bacteria and total bacteria plus total archaea were 0.5–2.8 and 0.1–2.7%, respectively, in the sands.

The 16S rRNA gene abundance of *M. nitroreducens*-like archaea was 2.1 × 10^4^ to 2.5 × 10^5^ copies g^−^^1^ dry sediment in the sands, while its abundance in the gravels was extremely low (<2.5 × 10^3^ copies g^−^^1^ dry sediment). Among these riverbeds, the River Nadder contained a relatively greater abundance of *M. nitroreducens*-like archaeal 16S rRNA genes (Fig. 7). The ratios of gene abundance of *M. nitroreducens*-like archaea to total archaea and total bacteria plus total archaea were 0.09–1.6 and 0.003–0.07%, respectively, in the  sands.

## Discussion

The potential for several pathways of AOM was detected in sandy riverbeds. Nitrite and nitrate are the dominant electron acceptors for AOM, although sulfate and ferric iron  both stimulated AOM in the Hammer Stream where methane concentration was highest (Table 1). Further, we provide evidence for the active involvement of *M. oxyfera*-like bacteria and *M. nitroreducens*-like archaea in nitrite- and nitrate/ferric iron-dependent AOM, respectively, in sandy riverbeds.

The significance of AOM in rivers is largely unexplored. For example, only molecular evidence of *M. oxyfera*-like bacteria has been reported previously, with no tracer measurements of activity [33–35]. Here, AOM activity was prevalent in the more reduced sandy riverbeds, whereas no activity could be detected in the more oxygenated gravel riverbeds. Both NO_2_^−^ and NO_3_^−^ stimulated AOM in all sandy riverbeds, and stimulation by NO_2_^−^ was greater than by NO_3_^−^ (Fig. 2). No obvious lag phase was observed for either nitrite- or nitrate-dependent AOM during the tracer experiments with a shorter preincubation time (6 h) and the activity (1.2–53.6 nmol CO_2_ g^−^^1^ [dry sediment] d^−^^1^) was similar to that (0.4–61.0 nmol CO_2_ g^−^^1^ [dry sediment] d^−^^1^) measured in experiments with longer preincubation times (2d) (Fig. 2), and the activity probably represented in situ potential. The rate of nitrite-dependent AOM measured in our rivers, particularly in the Hammer Stream, is considerably greater than that reported in lake sediments (1.8–3.6 nmol CO_2_ ml^−^^1^ sediment d^−^^1^) [23] (activity was determined after 3–4 weeks of incubation), or in wetland sediments (~10 nmol CO_2_ g^−^^1^ [dry soil] d^−^^1^; activity was determined within 1 day of incubation) [20, 21]. Zhu et al. [62] reported a potential nitrite-dependent AOM rate of 9 nmol CO_2_ g^−^^1^ (dry soil) d^−^^1^ in peatland after 3 months of incubation, with no activity being detected within the first 2 weeks. Furthermore, Deutzmann et al. [24] reported that the potential nitrite-dependent AOM rate, which was calculated from micro-sensor profiles, can be up to 40 nmol CH_4_ cm^−^^3^ d^−^^1^ in a lake water column. Here, a low porewater nitrite concentration was detected in our riverbeds (Fig. S1), which may be due to it being rapidly reduced either through AOM or anammox as, at times, nitrite is hard to detect at all [39]. The Hammer Stream sediments are known to support high in situ rates of nitrate reduction, with the majority being due to denitrification [41] and denitrification also dominates in the  rivers Nadder and Marden [39]. Therefore, the nitrite required for nitrite-dependent AOM in the sandy riverbeds may mainly derive from denitrification.

In lake and wetland sediments in which activity of nitrite-dependent AOM has also been reported, the sediments were probably impermeable silts and clays [20, 21, 23] where the transport of solutes is dominated by diffusion. In contrast, our gravel and sandy riverbeds are more permeable, allowing a greater exchange of solutes (NO_*x*_^−^) and dissolved methane [36] that could sustain a relatively higher potential of nitrite-dependent AOM. Although under maximum permeability in the gravels, the fast delivery of oxygen and low availability of methane appears unsuitable for the persistence of an active nitrite-dependent AOM community. In addition, ^13^C-carbon assimilation from ^13^CH_4_ was only measurable in the Hammer Stream; although we cannot exclude assimilation of any ^13^CO_2_ by other autotrophic organisms besides that assimilated directly by the *M. oxyfera*-like bacteria. Here, the CCE (8.2%) is lower than that previously reported for aerobic methanotrophs (~50%) in gravel riverbeds [32] but consistent with a CCE of 9% for an enrichment culture of *Candidatus* Methylomirabilis oxyfera [63], suggesting that the most reduced sediments of the Hammer Stream facilitate nitrite-dependent AOM.

Our nitrate-dependent AOM activity ranged between 0.5 and 20.0 nmol CO_2_ g^−^^1^ (dry sediment) d^−^^1^, lower than the nitrite-dependent AOM activity. This nitrate-dependent AOM activity is higher than that measured previously (within 24 h of incubation) in freshwater marsh sediments (0.4–1.2 nmol CO_2_ g^−^^1^ [dry sediment] d^−^^1^ [21]), but lower than that for paddy soils (e.g., 79.9 nmol CO_2_ g^−^^1^ [dry soil] d^−^^1^ [27]) where the activity was determined after 4 weeks of incubation following an obvious lag phase. It should be noted, however, that methane oxidation is not directly coupled only to NO_3_^−^ reduction in slurries amended with ^13^CH_4_ + NO_3_^−^ because both *mcr*A and *pmo*A genes were highly expressed in these slurries (Fig. 4) and some NO_3_^−^ may first be partially reduced to NO_2_^−^ (e.g., denitrification) and the NO_2_^−^ then used for methane oxidation.

We could measure comparatively little methane in the surface waters of our gravel (0.05–0.3 μM) and sandy rivers (0.3–5.1 μM) (Table S3), whereas methane was up to 100 times more concentrated in the sediment porewaters (~34.0 and ~354 μM, respectively, for the gravels and sands). Further, with more porewater methane data for the Hammer Stream and River Nadder (Fig. 1a), it is evident that the exponential decay in methane of 83–95% over the top 5 to 25 cm of sediment coincides with the in situ peak in nitrate reduction [41]. We have also measured aerobic methane oxidation in the sandy rivers here (except the Medway; Fig. S8) and 32 gravel rivers previously [64], with similar activity (1.7–47.4 nmol CH_4_ g^−^^1^ [dry sediment] d^−^^1^) to that of nitrite-dependent AOM. However, given that nitrate is typically more abundant than nitrite [65], it is more reasonable to use the AOM potentials measured with ^13^CH_4_ + NO_3_^−^ to estimate the role of NO_*x*_^−^-dependent AOM in attenuating methane emissions from sandy riverbeds. Accordingly, for the sandy riverbeds that are common in the UK (~26% of 9459 sites [37]) and probably beyond, we estimate that microbial methane oxidation is about 35% anaerobic and 65% aerobic. In more permeable and widespread gravel beds (48% of UK sites; [37]), however, methane oxidation is carried out almost exclusively by aerobic methanotrophs [32, 64, 66].

In addition to NO_2_^−^ and NO_3_^−^, SO_4_^2−^, and Fe^3+^ also stimulated AOM in the most reduced sediment from the Hammer Stream (Table 1), suggesting that high methane concentrations support a greater diversity of AOM pathways; although the potential rates for SO_4_^2−^ and Fe^3+^-dependent AOM were lower than NO_*x*_^−^-dependent AOM. The standard Gibbs free energies for nitrite- (−928  kJ mol^−^^1^) and nitrate-dependent AOM (−519.8 kJ mol^−^^1^) are greater than those for sulfate- (−16.6 kJ mol^−^^1^) and ferric iron-dependent AOM (−81.6 kJ mol^−^^1^) [67] and NO_*x*_^−^-dependent AOM is energetically more favorable. Thus, NO_*x*_^−^-dependent methanotrophs may have an advantage in using methane over the sulfate- and ferric iron-dependent methanotrophs.

Any methane still present below the dominant zone of nitrate reduction could also be used by sulfate- and ferric iron-dependent anaerobic methanotrophs, as here in the Hammer Stream. The Hammer Stream sediments are rich in Fe^2+^ (~178 µM) [41], which could be primarily from Fe^3+^ reduction, further suggesting that the Hammer Stream is a good environment for ferric iron-dependent AOM. The activity of sulfate- and ferric iron-dependent AOM (0.6–4.4 and 1.5–8.1 nmol CO_2_ g^−^^1^ [dry sediment] d^−^^1^, respectively) was at the lower end (3–36 nmol CO_2_ g^−^^1^ [dry sediment] d^−^^1^) of that reported in lake sediments, where AOM is probably coupled to sulfate and/or ferric iron reduction and NO_*x*_^−^ is comparatively scarce [16, 19].

To identify the AOM-mediating methanotrophs, the transcript abundance of *M. oxyfera*-like bacterial *pmo*A and *M. nitroreducens*-like archaeal *mcr*A was analyzed in conjunction with the ^13^C-tracer incubations. Our approach is therefore different from the most recent reports, in which AOM activity in lake and wetland sediments was linked to *M. oxyfera*-like bacteria or *M. nitroreducens*-like archaea only by detecting the presence of their specific 16S rRNA and/or functional genes [20, 21, 23, 24, 27]. The linear production of ^13^CO_2_ throughout the second set of tracer experiments, and prior to the gene expression experiments (Fig. 2), suggested that the microbial community did not change significantly during our incubations. Here, the addition of ^13^CH_4_ and NO_2_^−^or NO_3_^−^ significantly stimulated the expression of *M. oxyfera*-like bacterial *pmo*A (Fig. 4a), strongly suggesting that *M. oxyfera*-like bacteria are actively involved in nitrite-dependent AOM in our sandy riverbeds. The presence of *pmo*A genes with <93% nucleic acid sequence identity, compared to known species, has been taken to indicate novel methanotrophs [68]. Our recovered *pmo*A formed a distinct cluster only distantly related (89.4–90.5%) to the *pmo*A gene of *Candidatus* Methylomirabilis oxyfera [10], indicating that a potentially novel *M. oxyfera* cluster (or perhaps a novel *M. oxyfera* strain/species) is reducing methane emissions from sandy riverbeds. In addition to the *M. oxyfera*-like bacteria, some aerobic methanotrophs can use NO_2_^−^/NO_3_^−^ as alternative electron acceptors under oxygen limitation [69, 70]. Such aerobic methanotrophs may also play a role in methane oxidation here.

A significantly higher transcript abundance of *M. nitroreducens*-like archaeal *mcr*A was detected in slurries amended with ^13^CH_4_ + NO_3_^−^ (Fig. 4b), suggesting that these archaea are active and responsible for nitrate-dependent AOM in these sandy sediments. Also, the addition of Fe^3+^ stimulated the expression of *M. nitroreducens*-like archaeal *mcr*A (Fig. 4b) and, therefore, these archaea are probably responsible for ferric iron-coupled AOM in the Hammer Stream. Clusters I and II of the *mcr*A were both detected in the sediment incubations from the Hammer Stream and River Nadder amended with ^13^CH_4_ + NO_3_^−^, while cluster III could only be detected by addition of ^13^CH_4_ + Fe^3+^ to sediments from the Hammer Stream (Fig. S4). This indicates that clusters I and II were probably responsible for nitrate-dependent AOM, while cluster III was probably responsible for ferric iron-dependent AOM. Unlike *M. oxyfera*-like bacteria, which can only use nitrite, *M. nitroreducens*-like archaea may be more flexible, with the potential to use several electron acceptors.

No members of the marine anaerobic methanotrophs were detected in any of our samples and are unlikely to be major participants in sulfate-dependent AOM; though their abundance may have been too low to detect. Recent evidence suggests that *M. nitroreducens*-like archaea are involved in sulfate-dependent AOM in lake sediments [19] but here the addition of sulfate did not stimulate the expression of *mcr*A of *M. nitroreducens*-like archaea (Fig. 4b). However, we cannot exclude *M. nitroreducens*-like archaea performing sulfate-dependent AOM in our sandy riverbeds, because the specific *mcr*A primers used here may not have covered all lineages. Some 16S rRNA gene sequences of *M. nitroreducens*-like archaea recovered from the riverbeds were closely related (96–99% identical) to sequences reported from Lake Ørn (Fig. 6), where *M. nitroreducens*-like archaea are involved in sulfate-dependent AOM [19].

The 16S rRNA gene abundance of *M. oxyfera*-like bacteria (9.3 × 10^5^ to 1.5 × 10^7^ copies g^−^^1^ dry sediment) was similar to that previously reported from river (10^6^ to 10^7^ copies g^−^^1^ sediment) [34] and lake sediments (10^5^ to 10^6^ copies g^−^^1^ sediment) [59]. Here, the 16S rRNA gene abundance of *M. nitroreducens*-like archaea (2.1 × 10^4^ to 2.5 × 10^5^ copies g^−^^1^ dry sediment) was lower (*t*-test, *p* < 0.05) than that of *M. oxyfera*-like bacteria, which agrees with the lower nitrate-dependent AOM potentials, compared to nitrite-dependent AOM. The 16S rRNA gene abundance of *M. nitroreducens*-like archaea is higher than that in freshwater marsh sediments (10^3^ to 10^4^ copies g^−^^1^ dry sediment) [21], but lower than that in paddy soils (10^5^ to 10^6^ copies g^−^^1^ dry soil) [27].

In summary, we provide the first evidence of multiple active pathways of AOM driven by different electron acceptors in reduced riverbeds, including nitrite, nitrate, sulfate, and ferric iron. Among these, nitrite and nitrate-dependent AOM could be the most important in regulating methane emissions from more reduced sandy riverbeds, relative to more permeable and more oxygenated gravel beds. The diversity of AOM pathways is greatest where methane concentration is highest, suggesting a link between the diversity of AOM pathways and the availability of methane.

## Electronic supplementary material

Supplementary figures and tables
