# Supplementary material for: Active pathways of anaerobic methane oxidation across contrasting riverbeds
Source: ISME J. 2018 Oct 30;13(3):752–66. doi: 10.1038/s41396-018-0302-y (PMC6461903; doi:10.1038/s41396-018-0302-y)
Supplement: Supplementary file 1 — Supplementary figures and tables [file 41396_2018_302_MOESM1_ESM.docx]

**Supplementary figures and tables**

**Figure S1** Vertical porewater profiles of the (a) CH_4_ concentrations, (b) O_2_ concentrations, (c) NO_2_^-^ concentrations, (d) NO_3_^-^ concentrations and (e) NH_4_^+^ concentrations recovered *in situ*.

**Figure S2** Examples of ^13^CO_2_ production in sediments from the 2-4cm depth horizon amended with ^13^CH_4_ and different electron acceptors in the gravel riverbeds: (a) River Lambourn, (b) River Stour I and (c) River Stour II.

**Figure S3** Maximum likelihood phylogenetic analysis of the *M. oxyfera*-like bacterial *pmo*A cDNA (389 bp) at the nucleotide level. Bootstrap values > 50% (out of 1,000 replicates) are shown in front of respective nodes, and the scale bar represents 10% sequence divergence.

**Figure S4** Maximum likelihood phylogenetic analysis of the *M. nitroreducens*-like archaeal *mcr*A cDNA (186 bp) at the nucleotide level. Bootstrap values > 50% (out of 1,000 replicates) are shown in front of respective nodes, and the scale bar represents 10% sequence divergence.

**Figure S5** Relative abundance of the ten dominant bacterial phyla in sediments from the (a) Hammer Stream, (b) River Medway, (c) River Marden and (d) River Nadder.

**Figure S6** Relative abundance of the three dominant archaeal phyla in sediments from the (a) Hammer Stream, (b) River Medway, (c) River Marden and (d) River Nadder.

**Fig. S7** Correlation between the potential nitrite-dependent AOM rates and the 16S rRNA gene abundance of *M. oxyfera*-like bacteria.

**Figure S8** Potential rates of aerobic methane oxidation in the sandy riverbeds.

**Table S1** Precise location and bed-type of the study sites

| Rivers | Riverbed type | Latitude | Longitude |
| --- | --- | --- | --- |
| Hammer Stream  River Medway  River Marden  River Nadder  River Stour I  River Stour II  River Lambourn | Sand  Sand  Sand  Sand  Gravel  Gravel  Gravel | 51.14607  51.26798  51.31829  51.04385  51.15604  51.22574  51.44089 | 0.610196  0.518439  -1.86  -2.11182  0.828219  0.957806  -1.38661 |

**Table S2** The (q)PCR primers used in this study

| Primers | Sequence (5′-3′) | Specificity | Reference |
| --- | --- | --- | --- |
| 319f^a^  806r^a^  524f^a^  958r^a^  341f^b^  518r^b^  Arch967f^b^  Arch1060r^b^  qp1f^b^  qp1r^b^  641f^b^  834r^b^  cmo182^b^  cmo568^b^  McrA159f^b^  McrA345r^b^ | ACTCCTACGGGAGGCAGCAG  GGACTACHVGGGTWTCTAAT  TGYCAGCCGCCGCGGTAA  YCCGGCGTTGAVTCCAATT  CCTACGGGAGGCAGCAG  ATTACCGCGGCTGCTGG  AATTGGCGGGGGAGCAC  GGCCATGCACCWCCTCTC  GGGCTTGACATCCCACGAACCTG  CGCCTTCCTCCAGCTTGACGC  ACTGDTAGGCTTGGGACC  ATGCGGTCGCACCGCACCTG  TCACGTTGACGCCGATCC  GCACATACCCATCCCCATC  AAAGTGCGGAGCAGCAATCACC  TCGTCCCATTCCTGCTGCATTGC | Bacteria 16S rRNA  Bacteria 16S rRNA  Archaea 16S rRNA  Archaea 16S rRNA  Bacteria 16S rRNA  Bacteria 16S Rrna  Archaea 16S rRNA  Archaea 16S rRNA  *M. oxyfera* 16S rRNA  *M. oxyfera* 16S rRNA  *M. nitroreducens* 16S rRNA  *M. nitroreducens* 16S rRNA  *M. oxyfera pmo*A  *M. oxyfera pmo*A  *M. nitroreducens mcr*A  *M. nitroreducens mcr*A | (Fadrosh *et al.,* 2014)  (Fadrosh *et al.,* 2014)  (Pires *et al.,* 2012)  (Pires *et al.,* 2012)  (Muyzer *et al.,* 1993)  (Muyzer *et al.,* 1993)  (Cadillo-Quiroz *et al.,* 2006)  (Cadillo-Quiroz *et al.,* 2006)  (Ettwig *et al.,* 2009)  (Ettwig *et al.,* 2009)  (Schubert *et al.,* 2011)  (Schubert *et al.,* 2011)  (Luesken *et al.,* 2011)  (Luesken *et al.,* 2011)  (Vaksmaa *et al.,* 2017)  (Vaksmaa *et al.,* 2017) |

a-Primers used for PCR; b-primers used for qPCR

**Table S3** Physiochemical parameters of the surface water of the studied rivers

| Rivers | pH | Temperature  (℃) | Oxygen  (μM) | | Methane  (μM) | NO_2_^-^  (μM) | NO_3_^-^  (μM) | NH_4_^+^  (μM) |
| --- | --- | --- | --- | --- | --- | --- | --- | --- |
| Hammer Stream  Medway  Marden  Nadder  Stour I  Stour II  Lambourn | 7.0  6.8  7.8  8.2  6.3  7.3  8.1 | 11.0  17.6  14.3  14.1  18.6  14.6  11.6 | | 334.0  244.1  336.9  257.4  279.0  278.3  451.3 | 5.1  0.3  0.3  1.9  0.1  0.3  0.05 | 1.6  4.4  2.8  2.2  1.6  3.4  0.5 | 46.1  395.2  197.5  209.4  105.3  105.3  580.7 | 11.6  17.7  13.0  8.5  6.6  8.9  0.7 |

**Supporting References**

Cadillo-Quiroz H, Bräuer S, Yashiro E, Sun C, Yavitt J, Zinder S. (2006). Vertical profiles of methanogenesis and methanogens in two contrasting acidic peatlands in central New York State, USA. *Environ Microbiol* **8:** 1428-1440.

Ettwig KF, van Alen T, van de Pas-Schoonen KT, Jetten M, Strous M. (2009). Enrichment and molecular detection of denitrifying methanotrophic bacteria of the NC10 phylum. *Appl Environ Microbiol* **75:** 3656-3662.

Fadrosh DW, Ma B, Gajer P, Sengamalay N, Ott S, Brotman RM *et al.* (2014). An improved dual-indexing approach for multiplexed 16S rRNA gene sequencing on the Illumina MiSeq platform. *Microbiome* **2:** 6. doi:10.1186/2049-2618-2-6.

Luesken FA, Zhu B, van Alen TA, Butler MK, Diaz MR, Song B *et al.* (2011). *pmo*A primers for detection of anaerobic methanotrophs. *Appl Environ Microbiol* **77:** 3877-3880.

Muyzer G, de Waal E, Uitterlinden AG. (1993). Profiling of complex microbial populations by denaturing gradient gel electrophoresis analysis of polymerase chain reaction-amplified genes coding for 16S rRNA. *Appl Environ Microbiol* **59:** 695-700.

Pires AC, Cleary DF, Almeida A, Cunha A, Dealtry S, Mendonça-Hagler LC. *et al* (2012). Denaturing gradient gel electrophoresis and barcoded pyrosequencing reveal unprecedented archaeal diversity in mangrove sediment and rhizosphere samples. *Appl Environ Microbiol* **78:** 5520-5528.

Schubert CJ, Vazquez F, Losekann-Behrens T, Knittel K, Tonolla M, Boetius A. (2011). Evidence for anaerobic oxidation of methane in sediments of a freshwater system (Lago di Cadagno). *FEMS Microbiol Ecol* **76:** 26-38.

Vaksmaa A, Jetten MSM, Ettwig KF, Lüke C. (2017). *Mcr*A primers for the detection and quantification of the anaerobic archaeal methanotroph ‘*Candidatus* Methanoperedens nitroreducens’. *Appl Microbiol Biotechnol* **101:** 1631-1641.
